# Supplementary material for: Temporal Filtering to Improve Single Molecule Identification in High Background Samples
Source: Molecules. 2018 Dec 17;23(12):3338. doi: 10.3390/molecules23123338 (PMC6321103; doi:10.3390/molecules23123338)
Supplement: Supplementary file 1 [file molecules-23-03338-s001.pdf]

Supplementary Figures

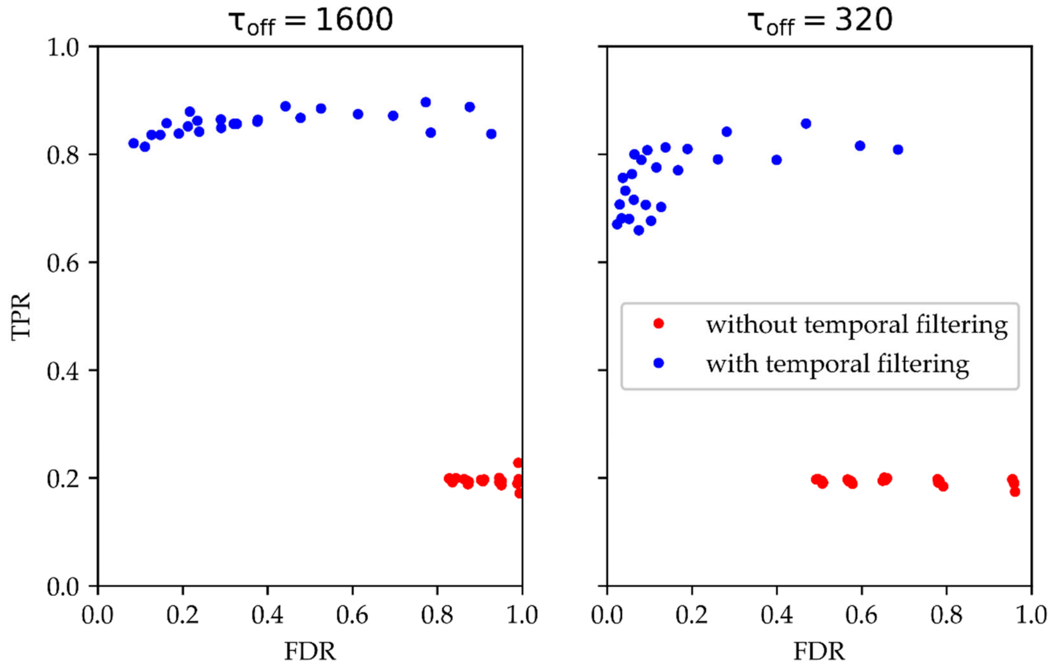

Supplementary Figure 1. Modified ROC Plot for different single molecule blinking scenarios. For the left panel we simulated molecules blinking at low frequency (τ_{on}=16; τ_{off}=1600), for the right panel we simulated blinking at high frequency (τ_{on}=16; τ_{off}=320); B was set to 500. The comparison shows data obtained without (red) and with (blue) temporal filtering. ω_T was set to 80%, analyzed with the algorithm by Gao et al [23].

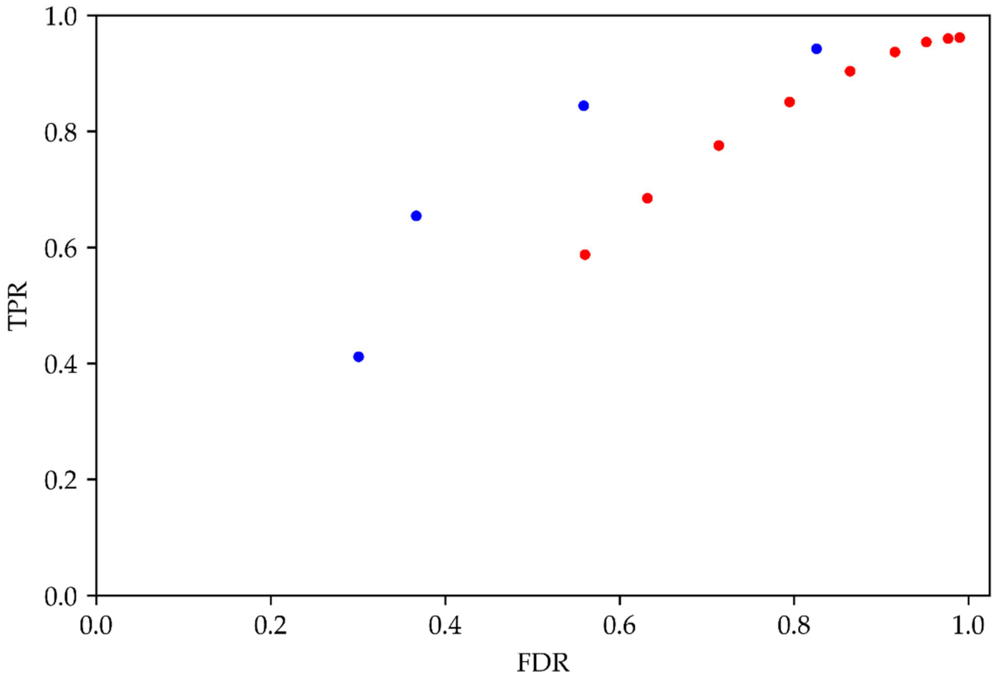

Supplementary Figure 2: Modified ROC Plot comparing temporal filtering (blue) and median filtering (red). For both simulations, we used τ_{on}=6, τ_{off}=600, B=500; ω_T was set to 80%. Data were analyzed with the algorithm by Gao et al [23].
